# Healthy Weight Regulation and Eating Disorder Prevention in High School Students: A Universal and Targeted Web-Based Intervention

**DOI:** 10.2196/jmir.2995

**Published:** 2014-02-27

**Authors:** Megan Jones, Katherine Taylor Lynch, Andrea E Kass, Amanda Burrows, Joanne Williams, Denise E Wilfley, C Barr Taylor

**Affiliations:** ^1^Laboratory for the Study of Behavioral MedicinePsychiatry and Behavioral SciencesStanford University School of MedicineStanford, CAUnited States; ^2^PGSP-Stanford PsyD ConsortiumPalo Alto UniversityPalo Alto, CAUnited States; ^3^Washington University in St LouisSt Louis, MOUnited States; ^4^Murdoch Children’s Research InstituteParkvilleAustralia

**Keywords:** healthy weight regulation, universal and targeted delivery, school-based intervention, prevention, adolescents

## Abstract

**Background:**

Given the rising rates of obesity in children and adolescents, developing evidence-based weight loss or weight maintenance interventions that can be widely disseminated, well implemented, and are highly scalable is a public health necessity. Such interventions should ensure that adolescents establish healthy weight regulation practices while also reducing eating disorder risk.

**Objective:**

This study describes an online program, StayingFit, which has two tracks for universal and targeted delivery and was designed to enhance healthy living skills, encourage healthy weight regulation, and improve weight/shape concerns among high school adolescents.

**Methods:**

Ninth grade students in two high schools in the San Francisco Bay area and in St Louis were invited to participate. Students who were overweight (body mass index [BMI] >85th percentile) were offered the weight management track of StayingFit; students who were normal weight were offered the healthy habits track. The 12-session program included a monitored discussion group and interactive self-monitoring logs. Measures completed pre- and post-intervention included self-report height and weight, used to calculate BMI percentile for age and sex and standardized BMI (zBMI), Youth Risk Behavior Survey (YRBS) nutrition data, the Weight Concerns Scale, and the Center for Epidemiological Studies Depression Scale.

**Results:**

A total of 336 students provided informed consent and were included in the analyses. The racial breakdown of the sample was as follows: 46.7% (157/336) multiracial/other, 31.0% (104/336) Caucasian, 16.7% (56/336) African American, and 5.7% (19/336) did not specify; 43.5% (146/336) of students identified as Hispanic/Latino. BMI percentile and zBMI significantly decreased among students in the weight management track. BMI percentile and zBMI did not significantly change among students in the healthy habits track, demonstrating that these students maintained their weight. Weight/shape concerns significantly decreased among participants in both tracks who had elevated weight/shape concerns at baseline. Fruit and vegetable consumption increased for both tracks. Physical activity increased among participants in the weight management track, while soda consumption and television time decreased.

**Conclusions:**

Results suggest that an Internet-based, universally delivered, targeted intervention may support healthy weight regulation, improve weight/shape concerns among participants with eating disorders risk, and increase physical activity in high school students. Tailored content and interactive features to encourage behavior change may lead to sustainable improvements in adolescent health.

## Introduction

Obesity is a growing problem for children and adolescents in the United States. The 2009-2010 National Health and Nutrition Examination Study data estimated the prevalence of obesity (body mass index [BMI] greater than the 95^th^ percentile for age and sex) as 18.4% for adolescents aged 12-19 years [1]; the rate has increased threefold in the last 30 years [2]. Late adolescence is a key developmental period for establishing lifelong health behaviors, as adolescent overweight can greatly affect health into adulthood [3]. Also, risk for eating disorder onset peaks in adolescence for girls [4], while research suggests that adolescent boys exhibit significant weight-related concerns [5]. Obesity and eating disorders may share risk factors, such as weight and shape concerns, loss of control eating, and unhealthy weight regulation behaviors [6]. Individuals who experience teasing about weight and shape are more likely to develop clinical and subclinical disordered eating symptoms, and adolescents who are overweight report significant weight and shape concerns [6]. These data indicate that, along with the shared risk profile, the overlap between overweight and disordered eating may be substantial and warrant targeted intervention. Finding ways to assess and treat the rise in obesity while sensitively addressing issues related to shape, weight, and body image in adolescents is a key public health goal, and one that has yet to be reached via traditional health programming. 

An Internet-based intervention model has unique benefits for obesity and eating disorder prevention, particularly for older adolescents. First, this model allows for universal and targeted intervention delivery. This is critical because by mid to late adolescence, risk factors may be more pronounced, and many adolescents may already be considered overweight or obese or have developed full-syndrome eating disorders. A universal prevention approach that targets cultural norms, policies, and encourages healthy weight regulation behaviors and positive body image among an entire population of students is essential, but may not be sufficient, for students at higher risk or struggling with more serious concerns [7]. Targeted (also referred to as selective) prevention interventions aim to reduce risk factors, in this case, those students already considered overweight who require information and tasks tailored to their unique needs [8]. Note that the term “targeted” as opposed to “indicated” prevention is used because of the notion that weight stabilization in adolescents may be more appropriate prevention strategy in this age group. Targeted interventions for weight gain and eating disorder prevention are also associated with larger effect sizes [9] and thus are a key component of a comprehensive program.

Second, the ability to seamlessly and anonymously provide a universal and targeted intervention can also help mitigate stigma, shame, and teasing, which are common psychosocial problems among adolescents who are overweight [10]. Singling out this group of students *in person* in a school environment is challenging and problematic for a number of reasons, including lack of appropriate resources and increased feelings of stigmatization, which can result in increased probability of dropout.

Third, Internet-based programs are relevant for an adolescent population and may therefore be more acceptable. Adolescence is a time of increased autonomy of food and activity choices and is associated with decreased participation in physical activity. Offering an intervention that complements adolescents’ normal routines (eg, using the computer) may increase adoption and retention. Further, this program does not seek to *increase* the overall time per day that adolescents spend online, but rather, encourage smarter and more effective use of technology to support healthy lifestyle behaviors [11]. Further, as it is delivered during classroom time, it does not add additional sedentary time.

Fourth, delivering interventions online transcends barriers associated with access to care, particularly among certain racial and ethnic minority groups who may not otherwise seek in-person services [12]. Online programs can also greatly enhance the sustainability of the delivery of evidence-based health interventions in school settings, by reducing the costs of providing tailored content for thousands of students at once. Recent research highlights how these programs increase versatility, require relatively low levels of professional support, and can be delivered in a variety of settings [13].

High schools provide an ideal setting in which to provide health promotion programs because of the ability to overcome health care disparities, access issues, and address sociocultural issues (eg, stigma) while meeting educational requirements [14]. However, issues with district policies and program implementation have negatively impacted health program delivery, despite an established need for such a curriculum [15]. Sustainable and cost-effective programs that easily integrate into an existing curriculum may increase adoption and sustainability.

This study describes StayingFit, a 12-session Internet-based program designed to build healthy habits and to support positive body image. Our model uses a universal and targeted approach, by giving youth and families personalized tools designed for use within a community-centered health program. StayingFit uses established principles of behavioral science (eg, self-monitoring and goal setting) to affect sustainable behavior change.

The purpose of this study was to test the feasibility, acceptability, and short-term efficacy of StayingFit for supporting healthy weight regulation and body image improvement among ninth-grade students. Secondary aims were to test for changes in psychosocial variables associated with StayingFit. We hypothesized that StayingFit would produce healthy nutrition (eg, increased consumption of “green” foods), lower levels of sedentary behavior, and reduce weight and shape concerns from baseline to post intervention.

## Methods

### Study Design

The StayingFit study was an uncontrolled feasibility study using a parallel, nonrandomized design. Twelve high schools were sent letters describing StayingFit and inviting collaboration in the research study. Of these, five schools expressed interest, and due to lack of direct funding for this study, two high schools were selected to participate based on ethnic diversity of the student body. Total enrollment for the San Francisco Bay Area school was 31% white, 4% Asian, 4% African American, 3% Pacific Islander, 0.5% American Indian or Alaska Native, and 2% other; 56% identified as Hispanic/Latino. At the St Louis site, students identified as 71% African American, 28% white, and 1% Asian; 1% of students identified as Hispanic/Latino. Note that for purposes of analysis in this study sample, data were analyzed according to the following racial categories: white/Caucasian, black/African American, multiracial/other, and did not specify. Ethnicity data for students identifying as Hispanic/Latino were also collected.

High school teachers and administration were approached in August 2010 to discuss implementing StayingFit through the physical education course. Enrollment began in February 2011. Approval from the Washington University and Stanford University Institutional Review Boards were obtained.

All ninth-grade students enrolled in physical education classes at the participating high schools during the 2010-2011 and 2011-2012 academic years were offered participation in the research study and completed StayingFit as part of their school’s physical education curriculum. Participation in the study was defined as agreement to undergo baseline and post assessments as indicated by active assent and parental consent by students and parents, respectively.

Measurement periods consisted of baseline and post-intervention assessments, occurring 1 week after the termination of the program. Track assignment was based on BMI percentile data. Students who were overweight (BMI percentile ≥85^th^ for age and sex; Centers for Disease Control [CDC], 2009) were assigned to receive the Weight Management (WM) intervention track, and students who were normal weight (BMI percentile <85^th^ for age and sex; CDC growth curves) were assigned to the Healthy Habits (HH) intervention track. Students were not informed of their track assignment in order to maintain confidentiality, anonymity, and reduce stigma. All students were told that they were participating in StayingFit*,* a program “for helping adolescents eat well, exercise and maintain a healthy weight, and feel better about their body image”.

### Recruitment

Teachers distributed consent forms to students during the first week of each semester, and parents and students were asked to read, sign, and return forms within 1 week. All participants were assigned unique identification numbers.

### Intervention

Teachers began delivering the weekly intervention in spring 2011, as part of their physical education course. All students were expected to finish one session per week as part of their class work, with any parts they were unable to complete assigned as homework. Teachers agreed to award class credit for completion of StayingFit sessions, to be determined at their discretion.

### StayingFit

The StayingFit program is a 12-session online program promoting healthy weight regulation and improved weight/shape concerns. StayingFit encourages adolescents to take an active role in their personal health attitudes and behaviors. A user-centered design process was used to develop StayingFit, and multiple prototypes, focus groups, and usability testing occurred over the course of several years prior to this study. The core content and structure of StayingFit was adapted from a set of validated programs: Student Bodies [16-18] and Student Bodies-BED [19,20]. Student Bodies is an online eating disorder prevention program for adolescent and college-age women that has been shown to significantly reduce risk, onset, and progression of eating disorders [18]. Student Bodies-BED (binge eating disorders) is a program designed for adolescents at risk for overweight, who may have symptoms of binge eating disorder. It has shown a significant effect on BMI z-score, weight and shape concerns, and binge eating behaviors [20].

The current 12-session program uses a unique screening algorithm to assign youth to individualized programs based on their weight status and eating disorder risk. The use of a validated eating disorder prevention program in the program “core” ensures that a unified message is provided to all students about the importance of developing a positive body image. The core of StayingFit also focuses on nutrition education, which uses the concept of “red” and “green” foods to teach students how to structure their diet [21]. Other key themes woven throughout include incorporating physical activity into daily activities and communicating with peers and family about health. New content in this version of StayingFit adds up-to-date nutrition and physical activity information, interactive online exercises, and modules about weight stigma and social pressures related to body image. See Tables 1 and 2 for a description of the intervention components and weekly themes. To ensure newly learned behaviors are sustained over time and across contexts, StayingFit is linked with family, peer, and school programming. Specifically, the program is delivered in classrooms in conjunction with existing health-oriented curricula, encourages parental and teacher involvement by providing educational newsletters and hosting informational meetings that complement program content, and can be readily integrated within related schoolwide health campaigns.

**Table 1:** StayingFit intervention components.

Component	Description
Sessions	Each StayingFit session includes 10-15 pages of online content, written at a 9^th^ grade reading level, designed to take approximately 30 minutes to complete.
Learning questions	At the end of each session, students are asked to answer questions about their learning that week. Questions assess knowledge, attitudes, behaviors, and self-efficacy related to specific skills taught in the session. To measure student engagement and enjoyment of the material, students rated the level at which content was helpful, interesting, and fun.
Food log	All students completed a food log, in which students indicated the number of separate servings of fruits, vegetables, other green foods (low fat, high nutrient foods), red foods (high fat, low nutrient foods), and soda they had on the day prior to completing the log. After students submitted the log, they were provided automated feedback related to goals (aligned with USDA guidelines) that they set in the first session of the program.
Meal size log	In the meal size log, students recorded meal times, sizes, and hunger before and after meals. Automated feedback was given based on number of meals and meal size, designed to encourage regular eating.
Physical activity log	Students are asked to record the type, frequency, and intensity of physical activity they completed over the previous week and set physical activity goals for the next week. Automated feedback about their exercise habits is provided.
Weight log (WM Track)	Students in the WM track may also complete a weight log, in which they recorded weekly weight and were provided cautionary feedback if they reported unhealthy weight loss or motivational feedback if they reported weight gain.
Hunger and fullness rating scale (Cohort 2)	A hunger/fullness scale ranging from 0 (starving/ravenous) to 10 (stuffed) was used to teach participants to be more attuned to their internal appetite cues. Participants were encouraged to monitor their hunger level throughout the day and to begin eating when their internal appetite signals reached a hunger level of 3, and to stop eating when they reached a 7.
Discussion board	All students were invited to comment on an anonymous discussion board, accessible 24 hrs a day, but primarily used during class to respond to questions related to program material.
Parent materials	Parents could receive weekly emailed or hardcopy newsletters that provided content coordinated with student sessions.
Teacher materials	Teachers were provided with “StayingFit Program FAQs” and invited to contact the research team with any questions.

**Table 2:** Weekly themes.

Week	Sessions covered	Topics/content
1	1	Introduce program, describe and educate about “red” and “green” foods, establish individual nutritional intake needs based on Choosemyplate.gov, introduce the concept of appetite monitoring including eating in response to moderate hunger and fullness cues
2	2	Introduce the importance of exercise & how exercise relates to weight management
3	3	Educate about lifestyle activities, set healthy and realistic exercise goals using the FITT principle, create a fitness plan
4	4, 5	4—Introduce the importance of regular eating, educate on reading nutrition labels, encourage cutting out high-calorie drinks 5—Binge eating: what it is and triggers, adding “forbidden foods” into your diet, review the concept of hunger/fullness monitoring, provide a mindful eating exercise
5	6	Body image, self-esteem, direct and indirect triggers of negative thoughts and feelings
6	7	Developing healthy routines with regards to eating, exercise and sleep, overcoming barriers to healthy eating
7	8, 9	8—Making healthy snack choices, serving sizes, why diets do not work and the negative effects of dieting 9—Learn about eating disorders, challenging negative thoughts and cognitive restructuring, feel-good body tips
8	10	Environmental factors that influence eating, eating healthy foods in “risky” situations
9	11, 12	11—Learning how not to participate in stigma about weight, ways to stay confident, strategies for dealing with teasing 12—Maintaining healthy habits in the long run, problem solving

### Tracks

StayingFit is divided into two parallel student tracks. The Healthy Habits (HH) track, for students below the 85^th^ percentile of age- and sex-adjusted BMI, describes the goal of the program as developing healthy habits related to nutrition and physical activity. The Weight Management (WM) track, designed for students above the 85^th^ percentile of age- and sex-adjusted BMI, emphasizes eating and exercise for weight maintenance. Track differences are primarily in the language used to describe the content and exercises, rather than the content itself. However, students in the Weight Management track also have access to an optional weight log (see below), if they choose to chart their weight each week.

### Sessions

The program pages included photos and interactive exercises designed to increase engagement. These exercises were free-text response questions, pop quizzes, fun facts, or matching games related to session content. Both tracks included interactive self-monitoring logs at the end of each session (see Table 1). Students were asked to complete three logs weekly. All students were invited to comment on an anonymous discussion board set up for StayingFit, on which the research team posted weekly questions related to program material. Students were notified that the discussion board is monitored daily by research team members for questionable content, and any potentially harmful posts are reported to faculty.

### Parent Materials

Parent newsletters that provided content coordinated with student sessions were made available in electronic and hardcopy formats.

### Teacher Materials

In order to reduce required time investment on the part of school staff, StayingFit was designed to be sustainable and easily implementable in classroom settings. To enhance program delivery, teachers were provided with “StayingFit Program FAQs” and invited to contact the research team with any questions.

### Schematic

**Figure 1:**
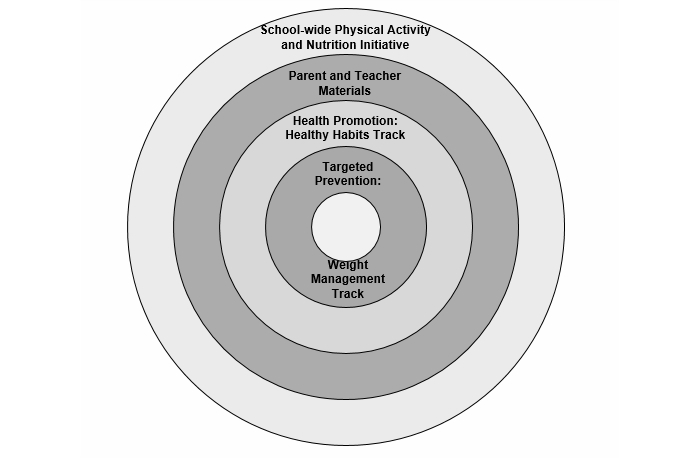
StayingFit Intervention schematic.

Figure 1 provides a schematic overview of the StayingFit intervention components in the context of the “levels” of intervention. As indicated in the diagram, the program was implemented in the context of school settings that participated in physical activity and nutrition education programs (ie, state and local school district requirements for education on these subjects). The StayingFit intervention directly addressed the levels of parents/teachers and individuals across two intervention levels: no/low risk (health promotion) and high-risk (targeted prevention). Students who required more intensive clinical services (eg, students with anorexia nervosa or with medical problems associated with severe obesity) were recommended to seek outside services.

### Programming and Privacy

HealthMunk LLC worked with the research team to program StayingFit and provide support throughout research trials. StayingFit is offered on a secured HIPAA-protected secure server and requires login and password credentials to access. Information is encrypted and no identifying information was stored for this study; only students’ usernames and passwords were entered into the online interface. Student usernames were chosen by the students, who were instructed not to include any potentially identifying words or phrases in their selection (eg, initials, portions of their name). Passwords were common words assigned by the research team. Students were instructed not to share their usernames or password with peers.

In order to ensure maximum privacy, teachers were not provided with student usernames but were given a list of student passwords, as many students misplaced their original password assignment slips. If students needed to retrieve their usernames, they were instructed to contact the research team.

### Measures

The research team visited classrooms at the beginning of each semester to present the program to the students and to help students complete the pre-assessment online via SurveyMonkey. At this time, students and teachers had the opportunity to ask questions. Students were asked to choose individual usernames and passwords to log on to the online interface and given contact information for the research team in case of further questions. The research team set up an email address for centralized communication about the study with parents, teachers, and students. The email was checked daily, and requests for contact were returned within 24 hours. Students completed post-intervention assessments at the end of 12 sessions. All assessments were completed during class time.

### Primary Outcomes: Anthropometric Measures

Teachers obtained height and weight 2 weeks prior to the baseline assessment using digital scales and wall measurements of height. At post treatment, students provided self-reported height and weight. Self-report at post treatment was more feasible than arranging for confidential weight and height measurements for all students and was more acceptable to students, teachers, and school administrators. The potential for inaccuracy in self-report anthropomorphic data was adjusted for by using a conservative approach in the analyses, as described in the Statistical Analysis section. BMI percentile and standardized BMI (zBMI) were calculated based on CDC growth charts for age and sex.

### Secondary Outcomes: Weight and Shape Concerns

Weight and shape concerns were assessed at baseline and post treatment using the Weight Concerns Scale (WCS) [22]. The WCS assesses worry about weight and shape, fear of gaining 3 pounds (1.36 kg), last time on a diet, importance of weight compared to other life areas, and feelings of fatness. The WCS is significantly correlated with the Eating Disorder Inventory total score (EDI; [23] and the body dissatisfaction subscale of the EDI [24]. The WCS has a test-retest value of approximately 0.85, a 1-year stability of *r=*.75 [25], and adequate predictive validity [22]. A cut-off score of WCS ≥47 had a sensitivity of 79% and a specificity of 67% for identifying adolescents who developed partial or full EDs [25].

The baseline and post measures also included selected items from the CDC Youth Risk Behavior Survey (YRBS; [26]) to assess nutrition and self-reported physical activity. Clinically meaningful cut-offs were used, based on weight loss recommendations from the Expert Committee, as endorsed by the American Academy of Pediatrics (CDC, 2011). Specifically, these were defined as consuming two or more servings of fruit or vegetables per day; engaging in 5 or more days of physical activity for at least 60 minutes; watching less than 2 hours of television per day; and playing less than 2 hours of video games per day. A cut-off of one or less soda drinks per day was used. The Center for Epidemiologic Studies Depression Scale (CES-D) [27] was used to assess mood.

### Statistical Analysis

Analyses were conducted using SPSS version 21.0 (SPSS Inc.). Data were screened for normality. There were large standard deviations in change in BMI percentile and zBMI, due to the use of self-report height and weight data at post intervention. We used a conservative approach of excluding participants with zBMI change greater than 1 standard deviation (SD) above and below the mean. This updated range was compared to the mean zBMI change observed between baseline and post intervention (ie, 0.25 change) in an efficacy trial of the Student Bodies-Binge Eating Disorder online intervention with adolescents [20], on which the StayingFit intervention was based.

Baseline values were imputed for missing post-intervention data (ie, n=71; 21%) on the main outcome variables: BMI percentile and zBMI. Chi-square analyses and *t* tests were used to examine baseline differences between participants in the two intervention tracks. Paired samples *t* tests and chi-square tests were used to examine differences from baseline to post test across the outcome variables. Regression analysis was used to examine the effects of intervention track on post-intervention assessment scores, controlling for baseline scores on the same measures. *P* values less than .05 were considered statistically significant; all tests were two-tailed.

## Results

### Participants

A total of 336 participants provided assent and their parents provided informed consent. These students were assigned an intervention track based on their BMI percentile. Of these participants, 225 students were assigned to the HH intervention track, and 111 students had been assigned to the WM intervention track. Table 3 provides baseline characteristics for the sample, separated by intervention track. For the total sample, the racial breakdown was as follows: 46.7% (157/336) multiracial/other, 31.0% (104/336) Caucasian, 16.7% (56/336) African American, and 5.7% (19/336) did not specify. 43.5% (146/336) of students also identified as Hispanic or Latino.

**Table 3:** Participant baseline characteristics, by intervention track.

Variable^a^		HH track (n=225)^b^	WM track (n=111)^b^	*P* value
Age in years, mean (SD)		14.3 (0.63)	14.3 (0.74)	.999
Female, n (%)		137 (60.9)	63 (56.8)	.480
Race, n (%)				
	White/Caucasian	91 (40.4)	13 (11.7)	<.001
	Black/African American	30 (13.3)	26 (23.4)	.029
	Multiracial/Other	94 (41.8)	63 (56.8)	.011
	Did not specify	10 (4.4)	9 (8.1)	.210
Ethnicity, n (%)				
	Hispanic/Latino	90 (40.0)	56 (50.5)	.079
BMI percentile, mean (SD)		52.8 (22.8)	94.5 (4.12)	<.001
zBMI, mean (SD)		0.04 (0.76)	1.74 (0.43)	<.001
WCS, mean (SD)		26.5 (20.2)	41.4 (19.8)	<.001
CESD, mean (SD)		12.7 (9.0)	15.6 (10.3)	.013
Ate fruit ≥2 times per day, n (%)		48 (24.8)	23 (25.3)	.894
Ate vegetables ≥2 times per day, n (%)		35 (18.1)	16 (17.6)	.370
Drank soda ≥1 time per day, n (%)		29 (15.0)	29 (31.2)	.003
Engaged in physical activity ≥5 days per week, n (%)		112 (58.0)	29 (31.8)	<.001
Watched television ≥2 hrs per day, n (%)		47 (24.3)	44 (48.4)	<.001
Played video games, ≥2 hrs per day, n (%)		47 (24.3)	24 (26.4)	.692

^a^Vegetables=green salad, potatoes, carrots, and other vegetables; Physical activity=at least 60 minutes of physical activity per episode.

^b^n is based on completion of measure at both time points.

### Change in Anthropometric Measures

From baseline to post intervention, BMI percentile for participants in the HH intervention track was stable (mean change –0.12 [SD 6.53]; *t*_224_=–0.27; *P=*.791), which was an expected finding. However, there was a significant decrease in BMI percentile for participants in the WM intervention track participants (mean change –0.50 [SD 1.49]; *t*_110_=–3.51; *P=*.001). There was no significant difference between the intervention tracks on BMI percentile scores at post intervention, controlling for baseline scores on the same measure (standardized beta=.02; *P*=.130).

A similar pattern of results was observed using zBMI. From baseline to post intervention, within-group zBMI change was stable for the HH intervention track participants (mean change –0.007 [SD 0.16]; *t*_224_=–0.68; *P*=.497), as expected. However, this change was significant for the WM intervention track participants (mean change –0.03 [SD 0.11]; *t*_110_=–2.84; *P=*.005). There was no significant difference between the intervention tracks on zBMI scores at post intervention, controlling for baseline scores on the same measure (standardized beta=–.007; *P*=.565).

### Change in Eating and Activity Behaviors

#### Overview

In total, 193 students (193/225, 85.8%) in the HH track and 91 students (91/111, 82%) in the WM track completed the YRBS measure at both time points.

#### Consumption of Two or More Servings of Fruit Over the Previous 7 Days

At post intervention, 61 participants (61/225, 31.6%) in the HH tracks and 26 participants (26/111, 28.5%) in the WM track had two or more servings of fruit over the previous 7 days. This was a significant increase from baseline to post intervention for participants in the HH track (χ^2^_1_=55.7; *P*<.001) and for participants in the WM track (χ^2^_1_=15.7; *P*<.001).

#### Consumption of Two or More Servings of Vegetables Over the Previous 7 Days

At post intervention, 41 participants (21.2%) in the HH track and 22 participants (24.1%) in the WM track had two or more servings of vegetables over the previous 7 days. This was a significant increase from baseline to post intervention for participants in the HH track (χ^2^_1_=15.3; *P*<.001). Change for participants in the WM track was not significant (χ^2^_1_=0.31; *P*=.752).

#### Consumption of Soda at Least Once per Day Over the Previous 7 Days

At post intervention, 34 participants (34/225, 17.6%) in the HH tracks and 24 participants (24/111, 26.4%) in the WM track had a soda at least once per day over the previous 7 days. This was a significant increase from baseline to post intervention for participants in the HH track (χ^2^_1_=13.3; *P*=.001) and a significant decrease for participants in the WM track (χ^2^_1_=10.5; *P*=.002).

#### Engaged in 60 Minutes of Physical Activity on at Least 5 of the Previous 7 Days

At post intervention, 85 participants (85/225, 44.0%) in the HH tracks and 31 participants (31/111, 34.1%) in the WM track engaged in at least 60 minutes of physical activity on at least 5 of the previous 7 days. This was a significant decrease from baseline to post intervention for participants in the HH track (χ^2^_1_=48.4; *P*<.001) and a significant increase for participants in the WM track (χ^2^_1_=14.9; *P*<.001).

#### Watched Television for 2 Hours or Less Over Previous 7 Days

At post intervention, 52 participants (52/225, 26.9%) in the HH tracks and 34 participants (34/111, 37.4%) in the WM track watched television for more than 2 hours per day over the previous 7 days. This was a significant increase from baseline to post intervention for participants in the HH track (χ^2^_1_=38.1; *P*<.001) but a significant decrease for participants in the WM track (χ^2^_1_=29.7; *P*<.001).

#### Played Video Games for 2 Hours or Less Over Previous 7 Days

At post intervention, 44 participants (44/225, 22.8%) in the HH tracks and 26 participants (26/111, 28.6%) in the WM track played video games for more than 2 hours per day over the previous 7 days. This was a significant decrease from baseline to post intervention for participants in the HH track (χ^2^_1_=53.4; *P*<.001) but a significant increase for participants in the WM track (χ^2^_1_=18.4; *P*<.001).

### Change in Psychosocial Variables

#### Weight and Shape Concerns

From baseline to post intervention, within-group change in weight and shape concerns was not significant for participants in the HH intervention track (mean change –1.55 [SD 12.9]; *t*_193_=–1.68; *P=*.095) or for participants in the WM intervention track (mean change –3.02 [SD 15.8]; *t*_90_=–1.82; *P=*.071). There was no significant difference between the intervention tracks on weight and shape concerns scores at post intervention, controlling for baseline scores on the same measure (standardized beta=.038; *P=*.327).

We also looked at the subset of participants who had elevated weight and shape concerns (ie, WCS score ≥47) at baseline. There was a significant decrease in weight and shape concerns among participants in the HH track (n=35; mean change –7.23 [SD 17.4]; *t*_34_=–2.46; *P=*.019) and among participants in the WM intervention track (n=36; mean change –7.69 [SD 15.4]; *t*_35_=–3.00; *P=*.005). However, weight and shape concerns remained above the 47 cut-point at post intervention among both groups.

#### Depressive Symptoms

From baseline to post intervention, there was a significant within-group increase in depression scores for participants in the HH intervention track (mean change 1.29 [SD 8.14]; *t*_167_=2.06; *P*=.041) but there was no significant within-group change for participants in the WM intervention track (mean change 0.67 [SD 10.3]; *t*_71_=0.55; *P=*.584). There was no significant difference between the intervention tracks on depression scores at post intervention, controlling for baseline scores on the same measure (standardized beta=.004; *P=*.932).

## Discussion

### Principal Results

An important objective of this study was to identify potential barriers to implementation and dissemination of a universal and targeted obesity and eating disorders risk reduction program in schools*.* Acceptability and feasibility of school-based implementation was high. Students and teachers reported satisfaction with program content and implementation and the intervention was inexpensive to deliver. This resulted in the school asking the investigators to offer the program again in subsequent years. Investigators provided assistance with computer problems, but the program was run nearly entirely by the teachers with no training. After the initial cost of program design, content and minor functionality changes could be easily completed.

As this was a pilot study, the primary results are related to short-term behavioral outcomes. BMI significantly decreased among students in the WM track. BMI was stable in the HH track, demonstrating weight maintenance. As has been noted previously, weight maintenance is often an appropriate goal for adolescent obesity prevention as weight stabilization is associated with numerous health benefits [28]. These results are consistent with those found in studies examining previous versions of StayingFit [19,20] and with other research suggesting that Internet-based weight management can be effective for adolescents [29].

The impact of StayingFit on weight and shape concerns was also consistent with previous research demonstrating that the intervention was effective in reducing weight and shape concerns among participants with elevated eating disorders risk. In this case, this finding suggests that StayingFit functioned appropriately as an eating disorder risk reduction intervention while simultaneously supporting health weight regulation. Reducing weight and shape concern among overweight participants is particularly impactful because these individuals are at highest risk for disordered eating behavior.

Eating behaviors also largely improved across tracks. Fruit consumption significantly increased in both groups and vegetable consumption increased significantly for HH participants, but results were not significant for participants in the WM track. The number of participants reporting daily soda consumption significantly increased in the HH group but decreased in the WM group. Similarly split results were observed for other health behaviors. Physical activity significantly decreased among HH participants but increased in WM participants. Increased rates of television viewing were reported by HH participants at post intervention, but WM participants reported a decrease. Interestingly, video game time decreased among the HH group but showed an increase in the WM group. This was not a randomized controlled study, so conclusions and generalizability about the true impact on behaviors are limited. However, the increase in physical activity and decrease in soda consumption and television time among adolescents in the WM track are encouraging. It seems that the StayingFit program was able to have a measurable impact on health habits, in some cases differentially between the two tracks, suggesting that universally delivered interventions can be successful in targeting specific risk behaviors among overweight adolescents.

The potential positive impact on physical activity in the WM track is particularly notable. Teachers, parents, and health practitioners have expressed concern about delivery of health programs online, suggesting that this mode of delivery encourages sedentary behaviors that can contribute to adolescent overweight. However, the research in this area does not support this assertion. In a recent meta-analysis, Internet-based physical activity interventions were shown to have significant effects on exercise behaviors [11]. In fact, these programs have shown promising results in populations identified as sedentary [30], significantly increasing steps per day in a study of overweight adults. These results suggest that online interventions can successfully translate into real-world behavior change. In this implementation, students offered StayingFit also participated in regular physical education classes and were given opportunities to put into practice the physical activity recommendations provided in the program.

However, physical activity decreased among the HH track, suggesting that further support for positive exercise habits is needed. It is possible that students in the HH track were less motivated to change health behaviors due to their normal weight status. Although content in StayingFit aimed to encourage physical activity among all participants, results suggest that this area of the program could be improved possibly capitalizing on competitions and more effective use of social media strategies. In future versions of StayingFit, we plan to include additional apps, games, and personal activity trackers that could further support healthy behavior change among all participants.

The results of this study add further support for the importance of developing Internet-delivered interventions that are dynamic and can be rapidly modified and tailored based on participant monitoring [31]. For example, in this study, participants recorded soda consumption in their self-monitoring logs. Increased soda consumption among the HH group could have triggered a “flag” for add-on supplementary content supporting targeted behavior change. The online format allows for easy intervention modification post hoc. However, to truly maximize the benefits of using technology, these “adjustments” should be made in real time.

### Limitations

This study was not intended to be an efficacy trial; hence, no control condition was used nor did randomization or long-term follow-up occur. Outcome data must therefore be interpreted with caution, and regression to the mean is highly possible with this study design. The study also represents implementation at two high schools, and future studies should examine the program feasibility and outcomes across multiple sites.

### Future Directions

Adolescent obesity continues to be an issue among US teenagers. Finding comprehensive ways to reduce adolescent overweight through assessment and education about eating, physical activity, and emotional distress may help alleviate health disparities and reduce the rates of common medical and psychiatric comorbidities.

A study utilizing an adaptive design could evaluate further iterative improvements, specifically in how to most effectively make use of in-program process data to tailor interventions [32]. The Internet-based format for health interventions has unique advantages that can be harnessed for positive impact at the individual and populations levels. The “virtual” nature of different program “tracks” allows students in the same classroom to be efficiently “screened” and assigned into intervention versions best suited for their needs. Real-time participant monitoring, meaning “in the moment” observation of program use, is also made more feasible by utilizing program use and self-monitoring journal data to ensure participant safety and provide referral when necessary. Online, moderated discussion groups for participants can leverage the strong influence of social networks to elicit healthy behavior change while still protecting student anonymity. Rather than being a static information source, Internet-delivered interventions harness technology to promote engagement, interactivity, and real-time monitoring and feedback.

While Internet-based approaches may offer a unique and important role in larger obesity and eating disorder prevention efforts, the intervention should not stop at the computer or mobile device. Rather, the technology should be used strategically to overcome limitations of person-based approaches for obesity and eating disorders prevention and facilitate connections with in-person social networks. Technology can aid in connecting individual-level interventions with broader socioecological systems and true universal prevention involving families, schools, communities, and policy change. Future studies should extend the socioecological framework of integrated obesity and eating disorders prevention through partnerships with community organizations and larger school and health systems.

### Conclusions

Results suggest that delivery of an Internet-based, universal, and targeted intervention is feasible and effective in a school-based setting. StayingFit appears to support healthy weight regulation, improve weight/shape concerns, and increase healthy food consumption in adolescents.

## Abbreviations

BMI: body mass index
CDC: Centers for Disease Control
CES-D: Center for Epidemiologic Studies Depression Scale
EDI: Eating Disorders Inventory
HH: healthy habits
HIPAA: Health Insurance Portability and Accountability Act of 1996
WCS: Weight Concerns Scale
WM: weight management
YRBS: CDC Youth Risk Behavior Survey
zBMI: standardized body mass index
